# Home-Based Primary Care’s Role in Supporting the Older Old During Wildfires

**DOI:** 10.1177/2150132719846773

**Published:** 2019-05-15

**Authors:** Tamar Wyte-Lake, Maria Claver, Rachel Johnson-Koenke, Aram Dobalian

**Affiliations:** 1 Veterans Emergency Management Evaluation Center (VEMEC), US Department of Veterans Affairs, North Hills, CA, USA; 2 Gerontology Department, California State University, Long Beach, CA, USA; 3 Denver-Seattle Center of Innovation (COIN), US Department of Veterans Affairs, Aurora, CO, USA

**Keywords:** community health, cost-effectiveness, primary care, access to care, emergency visits, home health, emergency preparedness, disasters

## Abstract

**Objectives:** There is limited understanding of how Home-Based Primary Care (HBPC) programs support their medically complex patients in event of a disaster. This study aimed to identify emergency preparedness protocols and procedures undertaken in advance of and due to the 2017 Northern California wildfires by staff of the Veterans Health Administration (VA) HBPC programs. **Methods:** This study examines the experiences and responses of two VA HBPC programs to the 2017 Northern California wildfires. Six phone interviews were conducted from July to August 2018. The interview protocol addressed agency preparedness policies and procedures, continuity of care after the wildfires, as well as facilitators and barriers to disaster response. **Results:** The total patient census of participating HBPC programs was 300. Neither HBPC program reported a loss of life due to the wildfires. Early patient preparedness, effective leadership support, and strength of program operating procedures emerged as key factors to effective response. **Conclusions:** Demand for home health care, like VA’s HBPC program, is projected to grow as the number of older adults and longevity increases. Emergency management efforts must likewise evolve to address the unique needs of these vulnerable patients in disasters. Understanding the program activities conducted by the VA HBPC programs in response to the 2017 Northern California wildfires can help improve the understanding of how VA and non-VA home-based care programs can be best integrated into resilience planning of local communities.

## Background

Individuals aged 85 years and older are the fastest growing segment of the US population.^1^ As the health care system tries to address how to optimally care for this medically complex population, one solution is to provide increased care directly in patients’ homes, keeping them home longer rather than transferring them to institutional care. The VA’s Home-Based Primary Care (HBPC) program is one example of this approach. The program selectively targets and provides longitudinal interdisciplinary care to veterans who present with complex chronic disease. The VA HBPC population has a mean age of 76.5 years.^2^ From 2006 to 2012, VA’s HBPC census tripled, growing from 7300 to 30 000, whereas VA-provided nursing home care rose only 20%.^3^ This significant increase is a singular example in the United States of the focus to keep more medically complex patients successfully living in their homes.

As older, more medically complex citizens remain in their home, there is an increased burden on the local community to serve them in a disaster. Traditionally, emergency management has been seen to have 4 phases, prevention-mitigation, preparedness, response, and recovery.^4^ Both The Joint Commission and Medicare have recognized the role home health programs have in supporting their patients’ emergency preparedness and response, and have thus increased these programs’ responsibility in preparing their patients for disasters.^5,6^

Each VA Medical Center has a HBPC program, which serves veterans needing care in the home and who are at heightened risk for morbidity and mortality during disasters such as Hurricanes Harvey and Irma. Previous studies have shown that the VA’s HBPC program is a key program in assessing and supporting the disaster preparedness of their patients.^7,8^ In data collected as part of a study conducted in 2016, 72% of HBPC Program Managers rated their current disaster preparedness programs as “somewhat robust” with 6% rating it as “not robust” and 22% rating it as “very robust,”^9^ demonstrating significant room for improvement in the perceived robustness of programs in their readiness to respond to disasters.

Wildfires that coursed through Northern California in the first part of October 2017 ravaged over 210 000 acres, forced 100 000 people from their homes, resulted in 185 hospitalizations, and caused 44 deaths.^10-13^ More than 100 000 veterans lived in the affected counties. Multiple VA Hospital Incident Command Centers were activated in Northern California due to the widespread wildfires.

There is limited understanding of the role that home care programs actually play during real-world emergencies and disasters. This study aimed to assess the role of VA HBPC programs in supporting their patients in advance of, during, and after the 2017 Northern California wildfires.

## Methods

This study used qualitative content analysis to examine the experiences of VA HBPC programs in their response to the Northern California wildfires.

### Sample

The 2 VA HBPC sites affected by the Northern California wildfires were included in this project. In both cases, the HBPC program’s affiliated VA Medical Center was not in the affected Fire Zone, but an affiliated Community-Based Outpatient Clinic (CBOC) was. The HBPC Program Director and affiliated practitioners from the 2 HBPC programs were invited to participate in this study.

### Data Collection Methods

Data were collected through telephone interviews with practitioners at each of the HPBC sites. The 30- to 60-minute interviews were semistructured, meaning that an interview guide was used to organize the interview, while allowing the interview to cover topics emerging from respondents that may not have been specific items on the interview guide. The HBPC Program Director was the first point of contact at each site. The director then identified additional appropriate practitioners who were invited to participate. The interview protocol was structured to follow the VHA Office of Emergency Management’s Comprehensive Emergency Management Program (CEMP),^4^ and separately addressed agency preparedness policies and procedures (ie, mitigation and preparedness), continuity of care after the wildfires (ie, response and recovery), as well as facilitators and barriers to disaster response.

### Analysis Plan

Interviews were audio recorded with the permission of the respondent, and the interviews were transcribed verbatim. Transcripts were analyzed using qualitative content analysis based on a priori codes derived from the interview guide, as well as through the inductive development of codes based on the content of completed interviews. Authors TW and RJ independently coded the data, discussed discrepancies, and resolved those by consensus. The VA Greater Los Angeles Healthcare System Institutional Review Board (Los Angeles, CA, USA) approved this study.

## Results

Six team members, including program leadership and practitioners, from 2 Northern California VA HBPC programs impacted by the 2017 Wildfires participated in this research study. The respondents included site Program Directors, as well as representatives from Nursing, Social Work, and Program Support. The total patient census of participating HBPC programs was 300. Neither HBPC program reported a loss of life due to the wildfires. Four key themes emerged from the analysis of the resulting data: (1) Role of HBPC, (2) Leadership Support, (3) Learning Experiences, and (4) Recommendations. We have structured the results to be presented in 2 of the most salient emergency management categories: Preparedness Activities and Response Activities, with the 3 themes highlighted throughout.

### Preparedness Activities

#### Role of HBPC

Both programs reported engaging in a series of regularly occurring general disaster preparedness activities. These activities ranged from a cursory review of handbook materials about emergency preparedness provided to the patient, to conducting (quarterly or annual) emergency drills with patients. These drills included calling patients and verifying that they have their preparedness items at home. One program director, in noting the benefit of conducting the regular drills, highlighted both patient expectations due to regular drilling as well as provided an example of the familiar relationship established between the HBPC program staff and their patients,

I can remember the first day [of the Wildfires], when we had the command staff meeting, I remember the first day when they asked me a question and they said, oh are you calling your patients? I said are you kidding? Our patients expect us to call them because we do it quarterly. [They know] what we’re going to ask them.

Additional preparedness activities included verifying patient contact and next-of-kin or local emergency contact information at every patient visit, aiming to always have a 30-day supply of medication in the home, and making sure patients dependent on oxygen have emergency supplies, such as compressed oxygen cylinders. One site indicated that their oxygen supplier is contracted to bring oxygen to patients who are evacuated to a shelter.

#### Leadership Support

The extent of preparedness activities seemed to be, in part, related to the presence of a “disaster preparedness” champion at a site. One of the program sites had a program director who was highly involved in emergency preparedness activities at their facility, and as such, had a number of practices in place to facilitate a tightly organized response in the event of an emergency. These included having a weekly updated printed list of patients including home addresses and telephone numbers sorted by zip code to be taken home by practitioners and program leadership, so that, even if loss of electrical power renders computer files inaccessible, it is easy to ascertain whether any patients live in an emergency zone. Additional practices include ensuring all staff review the emergency preparedness standard operating procedures annually; stocking HBPC, government-owned cars with basic emergency kits; and signing up to receive text alerts from community and non-VA organizations such as CAL FIRE, regarding potential incidents in the region.

#### Learning Experiences and Recommendations

Staff identified that calling patients during annual or quarterly drills was very helpful because it provided an opportunity for staff to spend time with patients specifically reviewing emergency preparedness. They identified that this type of care can often be overlooked during a regular visit. After the wildfires, some staff noted an increased emphasis on preparedness with their patients. A nurse case manager who himself evacuated due to the wildfires noted,

We do [review emergency preparedness] on the first initial visit. I will notice a change in my behavior . . . after the fire I, each time I do an annual assessment I remind patients of that [emergency preparedness] packet they have or bring out those things about earthquake preparedness, having water. We always do that, their smoke detectors, make sure they have extinguisher. But now I really push heavy on the water.

Additionally, staff reported signing up for text alerts from CAL FIRE, and the Director at one of the sites added the CAL FIRE contact information to the home patient list as a backup as CAL FIRE was one of the most essential tools for notification and tracking of the numerous wildfires across the region.

### Response Activities

#### Role of HBPC

Leadership at both facilities underscored that the role of HBPC at the time of disaster does not include the physical evacuation of patients. Actual evacuation notwithstanding, there are numerous activities HBPC programs did report undertaking with their patients after their communities were affected by the wildfires.

All respondents noted that the first action taken after notification of the onset of the wildfires was to call their patients. In one case, a respondent who was evacuated from his own home in the middle of the night began calling patients as soon as he and his family were safely evacuated. As noted by one respondent,

there is a definite [mission] from our service chief as far as the clinic goes back . . . that we’re going to make sure our patients are always taken care of.

The postfire phone calls, which continued to be conducted with each patient daily until the clinics reopened, included questions assessing the patient’s medical and mental health status, identifying any breathing problems, and ensuring the patients had sufficient meds. If a patient was in the evacuation zone, questions would focus on finding out whether the patient evacuated his or her home, and if so, whether he or she found shelter. Although HBPC programs are unable to advise patients as to the location of preestablished shelters, one of the sites noted that practitioners visited their patients at the shelters. As the job of HBPC is to visit patients at their home, the shelter was considered to be the patient’s temporary home.

#### Leadership Support

HBPC staff found themselves in evacuation zones, displaced out of their homes, or, if able to remain in their homes, staff could be at home without power. Staff living in these regions found a greater need to rely on leadership and staff at their affiliated VA Medical Center (VAMC) for support. The extent of support seemed to be dependent on the robustness of the preparedness policy in place at each facility. As there seemed to be no formal plan in place for contacting patients at one of the sites, staff from the affiliated Community Based Outpatient Clinic (CBOC) and VAMC both independently contacted their patients in an ad hoc manner. It took a few days for the staff from the CBOC and VAMC to coordinate their phone calls. As noted by a respondent,

So a lot of us were . . . starting to make phone calls, checking on people we were most concerned about . . . It was very independent, it didn’t feel like it was organized, like who’s doing what . . . So ultimately I would find out, like if I’d call somebody, I’d have a person say oh, I already got a phone call from such and such a person.

This duplication and lack of coordination was noted with frustration by multiple staff, who suggested that a more coordinated effort by leadership would have helped reduce their own stress due to the impact of the wildfires on both themselves and their patients.

Knowing when to initiate a program response is an essential component of effective response activities. As HBPC’s role in providing home-based care falls outside the typical activities of the affiliated VAMC, special considerations may be needed to ensure that the VAMC’s emergency management plans also address the needs of the patients served by HBPC programs. Nevertheless, it is important that the needs of the HBPC program and its patients not be separated from the VAMC’s emergency management plan. As noted by one of the Program Directors,

I usually start my own program response and I’ll tell you why. Because facility emergency management, they are most concerned with the patients that are in our building, that are under our roof. They are most concerned about the patients, the employees in that setting and the infrastructure, the building. They are not, it’s not even on their radar usually what’s happening in the community . . . [and] we’ve started this, if an incident command post is formed, please make sure we are included.

#### Learning Experiences and Recommendations

Coordinating staff in the response period emerged as an important learning experience. Staff at one site started a texting chain among themselves to communicate with each other. However, staff reported that the texting chain became cumbersome as messages proliferated without clear goals. Staff recommended that CBOC staff immediately check in with the affiliated VAMC after an event, because the parent facility more consistently had access to computers and that would have simplified coordinating the staff response.

The ability for staff to telework became important in following up with patients after the wildfires. Teleworking allowed staff to follow up with their patients no matter where they were located and no matter whether the announcements of the wildfires came during regular operating hours. For both clinical and administrative staff who were unable to telework, learning about a fire over the weekend or trying to coordinate a response plan became extremely challenging, requiring the involvement of administrative support staff from one outpatient clinic having to contact staff from another clinic, at home, on a weekend, in order to get access to patient information stored behind the VA firewall.

## Discussion

The recently released Fourth Annual National Climate Assessment reports the impact of climate change creating new risks and exacerbating existing vulnerabilities in communities across the United States, presenting growing disaster-related challenges to human health and safety.^14^ Past disasters have shown that advanced age is one of the most significant factors for increased mortality, both during and in the first year after a disaster event.^15,16^ The population served by the VA’s HBPC program serves as an example of community-dwelling elderly who are at heightened risk during and after disasters. In this study, we sought to examine the VA’s HBPC program’s preparedness and response activities related to the 2017 Northern California wildfires to identify barriers to and best practices for similar home health programs during disasters.

The 4 phases of emergency management include mitigation, preparedness, response, and recovery. Our data shows the VA HBPC programs have a vital role in supporting their patients through each of these phases. Each of these phases poses distinct challenges for homebound populations such as those served by the HBPC program. For example, research has found that the “older old” often struggle with mitigation and preparedness.^17^ Having the resources to prepare, knowing how to take action steps toward mitigation and preparedness, and actualizing these steps are all challenges for this segment of the population, especially when they have limited financial resources. This is true even when a caregiver is present.^18^ The fact that HBPC programs provide medical care directly in patients’ homes offers HBPC staff the opportunity to tailor mitigation and preparedness planning directly to the complex needs of their patients. Moreover, all respondents noted that consistent follow-up with patients about mitigation and preparedness is as an essential piece of their programs’ preparedness protocols.

Although HBPC programs are not first responders,^7^ a core principle for HBPC staff is ensuring their patients’ safety and well-being. As isolation and lack of social support are significant concerns with this population,^19^ the role of HBPC in response and recovery becomes that much more critical. By tracking patients postdisaster, the HBPC program strengthens the health resilience of many of the community’s most vulnerable individuals. Disasters are highly likely to exacerbate chronic conditions in older adults and can thus increase emergency room visits.^17^ Building the disaster resilience of HBPC patients can thereby also yield substantial cost-savings in foregone morbidity.

With the numerous tasks involved in supporting patients before, during, and after a disaster, strong leadership support for home health’s disaster responsibilities is invaluable. A disaster preparedness champion who is aware of the unique vulnerabilities of the HBPC population can not only help a site develop strong mitigation and preparedness activities, but can also facilitate early, organized response that is critical to decreasing the disaster-related risk of morbidity and mortality among HBPC patients. A strong disaster preparedness and response standard operating procedure, combined with regular drills, could also ameliorate the frustration some staff reported with the lack of communication, coordination, and confusion regarding their role during disasters.

This study has limitations. First, due to the qualitative research design and the small sample size of VA programs, the study findings cannot be generalized to non-VA home health providers or all VA HBPC programs. Additionally, although efforts were made to include all HBPC staff, some staff declined to participate because they did not feel ready to talk about their experience during the wildfires. Finally, the sites affected by the wildfires are also in a region highly susceptible to earthquakes, and therefore their level of preparedness activities might differ from that of VA HBPC programs that are not located in similar disaster-prone regions. Future research should examine whether programs located in disaster-prone regions are more prepared than others and, if that is correct, how to ensure adequate preparedness in regions that are less frequently impacted by disasters. Furthermore, future research should also include the outcomes of the HBPC household emergency preparedness interventions, that is, probing into whether these patients are able to reside in their home or in the place of refuge safely and without an exacerbation of their chronic illness.

## Conclusion

As our population ages and more individuals continue to live in their homes despite declining health, communities’ emergency management efforts need to evolve to address their needs. Home health care programs such as the VA’s HBPC program have the potential to play a unique role in their patients’ lives but are not always fully prepared to do so. Understanding the barriers and facilitators to the HBPC response to the 2017 California wildfires and identifying best practices from such events, provides both the VA’s HBPC program and non-VA home health programs with a roadmap for better serving their vulnerable patients during disasters.
